# Brain FADE syndrome: the final common pathway of chronic inflammation in neurological disease

**DOI:** 10.3389/fimmu.2024.1332776

**Published:** 2024-01-18

**Authors:** Khalid A. Hanafy, Tudor G. Jovin

**Affiliations:** ^1^ Cooper Neurological Institute and Cooper Medical School at Rowan University, Camden, NJ, United States; ^2^ Center for Neuroinflammation at Cooper Medical School at Rowan University, Camden, NJ, United States

**Keywords:** microglia, IL-6, PASC, vagus, inflammation, depression, COVID, stroke

## Abstract

**Importance:**

While the understanding of inflammation in the pathogenesis of many neurological diseases is now accepted, this special commentary addresses the need to study chronic inflammation in the propagation of cognitive Fog, Asthenia, and Depression Related to Inflammation which we name Brain FADE syndrome. Patients with Brain FADE syndrome fall in the void between neurology and psychiatry because the depression, fatigue, and fog seen in these patients are not idiopathic, but instead due to organic, inflammation involved in neurological disease initiation.

**Observations:**

A review of randomized clinical trials in stroke, multiple sclerosis, Parkinson’s disease, COVID, traumatic brain injury, and Alzheimer’s disease reveal a paucity of studies with any component of Brain FADE syndrome as a primary endpoint. Furthermore, despite the relatively well-accepted notion that inflammation is a critical driving factor in these disease pathologies, none have connected chronic inflammation to depression, fatigue, or fog despite over half of the patients suffering from them.

**Conclusions and relevance:**

Brain FADE Syndrome is important and prevalent in the neurological diseases we examined. Classical “psychiatric medications” are insufficient to address Brain FADE Syndrome and a novel approach that utilizes sequential targeting of innate and adaptive immune responses should be studied.

## Graphical Abstract

Brain FADE Syndrome. Pictorial representation of the issues that patients face from disease pathogenesis to finding the correct type of physician to deal with Brain FADE Syndrome.
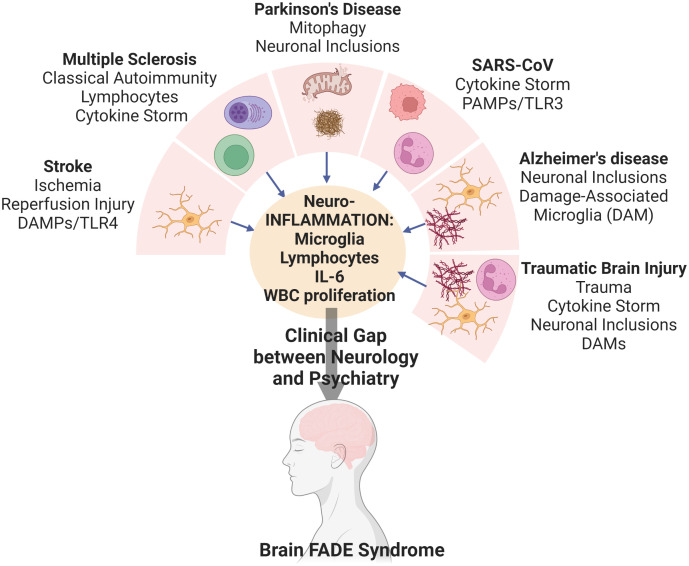

## 1 Introduction

The role of inflammation whether via damage-associated microglia, increased blood brain barrier permeability, or increased complement activity has a critical role in the pathogenesis of both cerebrovascular and neurodegenerative diseases (1). The brain, long thought an immunologically privileged organ, is now seen as immunologically active as the spleen with pseudo germinal centers, and resident innate and adaptive immune cells. With this understanding, the role of neuroinflammation has come to the forefront as an underlying etiology of neurological disease; however, the “psychiatric endpoints” have not received their due recognition.

While the neurological diseases of *depression*, *fatigue, and brain fog* affect a majority of patients with stroke, Alzheimer’s, Parkinson’s, multiple sclerosis, traumatic brain injury, and “long COVID,” the role of chronic inflammation in mediating these very important neurological diseases have not been well-studied. We named this combined endpoint of depression, fatigue and brain fog from chronic inflammation as Brain FADE syndrome; cognitive Fog, Asthenia, and Depression Related to Inflammation. We posit that chronic inflammation is the final common pathway that connects all neurological diseases, regardless of initial insult, to Brain FADE Syndrome.

## 2 Stroke

Estimates of Brain FADE post-stroke vary from 20-70%, based on a conglomerate of all of these symptoms (2). While active attempts to uncover the individual contributions of fatigue, fog, and depression in classical autoimmune diseases like multiple sclerosis or novel diseases like Long Covid are underway (3, 4), the endpoint is generally combined in stroke as depression. The FLAME trial was the first randomized placebo-controlled trial (RCT) that indirectly addressed Brain FADE after stroke (5). While the hypothesis of using fluoxetine in stroke was based on increasing excitatory neurotransmitters to promote neurogenesis, fluoxetine is more commonly used in the treatment of depression (6). In this small study of 118 patients both depression and motor recovery were significantly improved by fluoxetine treatment. Due to the impressive effects of fluoxetine, but the small sample size, an attempt to replicate the findings in large, collaborative studies was performed: the FOCUS, EFFECTS, and AFFINITY trials (7–9). The FOCUS trial was an RCT that enrolled approximately 3,000 stroke patients in the UK and found no difference in functional outcome, and interestingly, the treated arm saw less depression, but an increase in bone fractures. Similar studies were carried out in Sweden with the EFFECTS trial and AFFINITY between Australia, Vietnam, and New Zealand. Both RCTs found similar results to FOCUS in that there was no change in functional outcome, with a decrease in new depression; however, both trials also demonstrated more falls, more bone fractures, and more seizures.

Perhaps the reason that fluoxetine not only failed to improve motor outcomes, but was potentially harmful causing seizures, falls, hyponatremia, and fractures lies in the underlying pathophysiology of stroke. Fluoxetine does not address the underlying inflammatory etiology of chronic stroke symptoms, and potentially exacerbates the vascular etiology based on known mechanisms of this selective serotonin reuptake inhibitor. A focus on anti-inflammatory signal transduction may prove beneficial in the development of novel treatments for Brain FADE Syndrome.

The best studied “anti-inflammatory” agents in stroke with any data on depression and fatigue are the NSAIDs and statins. While SPARCL revealed no significant change in functional stroke outcomes, subsequent analysis of these data and others revealed mixed effects of statins on depression and fatigue (10). In a retrospective study in the Danish population, a study of over 300,000 people, the use of NSAIDs or ASA revealed a decreased risk of early depression after stroke, with the opposite effect on chronic depression. Conversely, the opposite temporal relationship was found with statin use alone (11). Another database study out of Taiwan with 11,000 patients demonstrated an increase in depression with statin use, while a smaller prospective study showed a slight decrease in depression (12, 13). Interestingly, the strongest link between depression, inflammation and statins is via the interleukin 6 (IL-6) pathway. Increases in IL-6 and IL-18 have been associated with depression after stroke, but no relationship to statins were drawn in these studies (14, 15). A weak causal relationship was established with a prospective study involving 400 Taiwanese patients indicating that statin-treated patients produced less IL-6 and had lower rates of depression, supporting an inflammatory hypothesis for depression.

The first anti-inflammatory RCTs in ischemic stroke were the SAINT trials where the free radical scavenger NXY-059 was used (16, 17). While SAINT I showed efficacy of the scavenger at reducing disability at 90 days, the repeat trial with twice as many patients failed to reproduce these effects. After initial evaluations in young, healthy male animals, further studies should be performed in females, aged animals, and animals with comorbid conditions such as hypertension, diabetes, and hypercholesterolemia. Although STAIR criteria for optimal research design in pre-clinical studies had recently been established, the pre-clinical studies did not include females, aged animals, and animals with comorbid conditions such as hypertension, diabetes, and hypercholesterolemia (18, 19). These are some of the reasons that NXY-059 may have failed. Importantly, NXY-059 may have influenced Brain FADE, but these outcomes were not measured.

The ESCAPE NA1 trial was another RCT with a multitude of pre-clinical studies supporting the concept that interfering with the interaction between post-synaptic density 95 and the NMDA receptor would prevent both the neurotoxicity induced by calcium influx as well as that from NOS II activation (20–22). The ESCAPE NA1 trial also failed to demonstrate any neuroprotection, yet again, no mention of any of the components of Brain FADE in the outcomes measured in the RCT or tested in the pre-clinical studies. While the preclinical studies gained much fame, the extent to which they were replicated in females, aged, and comorbid mice may have also lead to issues with the translation to human stroke.

A more direct approach studying the relationship between stroke and inflammation was taken in the ACTION trial where an inhibitor of leukocyte-endothelial interaction, effective in the treatment of multiple sclerosis, was tested (23). Although the natalizumab trial did not find any significant effects of infarction volume or functional outcome, depression and fatigue were not analyzed. The failure of ACTION may have many components, including timing and dosage, but the most striking difference is duration of treatment. Patients in ACTION received 1 dose of the drug within 9 hours of stroke, whereas in RCTs involving MS patients, the drug was given once per month for over 2 years (24, 25). Furthermore, natalizumab seemed to have remarkable efficacy in reducing fatigue and depression in MS patients; however, these endpoints were not studied in ACTION (26–29).

The newest anti-inflammatory agent to be bridged from bench to the bedside is a Toll-like receptor 4 (TLR4) aptamer. The toll like receptors are evolved to prevent prokaryotic attack of eukaryotic cells. Canonical TLR ligands are known as pathogen associated molecular patterns (PAMPs) that recognize and respond to specific common antigens on viruses, fungi, or bacteria. Similarly, danger associated molecular patterns (DAMPs) are endogenous molecular patterns that signal damage to the body like heme or mitochondrial membrane fragments (30, 31). Although the ligand to TLR4 in ischemic stroke is not as well-established as in hemorrhagic stroke, conditional and whole body knockouts of TLR4 in all models of stroke have demonstrated profoundly neuroprotective effects (32–36).

To translate this understanding to human ischemic stroke, aptamers or short sequences of nucleic acids that bind to inhibit and TLR4 have been developed. Aptamers, in theory, should result in a reduced inflammatory response due to lack of protein antigens and sequences that are not recognized as DAMPs by other toll like receptors. Preliminary studies demonstrate that the drug is safe and more importantly, Brain FADE is being investigated as an endpoint in future clinical trials (36–38).

Thus, future investigations into reducing neuroinflammation should include Brain FADE as an endpoint. Even if the pharmaceutical intervention does not show efficacy against “functional” outcome, as perceived by the physician, treating Brain FADE may improve the patient’s perception of the outcome. Ultimately, that is the goal of any physician-patient interaction. This concept of the patient’s opinion of a functional outcome is not entirely novel and has been used in novel metrics such as the utility-weighted modified Rankin Score (39).

## 3 Multiple sclerosis

Multiple sclerosis, on the other hand, is the archetypal model of cerebral inflammation, and synonymous with neuroimmunology. As such, it is not surprising the Brain FADE has been studied extensively; fatigue (40), fog also known as “cog fog” (41), and depression (42) are estimated to affect approximately 37–78% (43), 34–65% (44), and 5–59% (45) of patients, respectively. Of note, not only have each of these elements of Brain FADE syndrome been individually studied in MS, but the idea that they are part of the final common pathway due to the inflammation in MS, and are observed in aggregate, has also been studied in such detail that 252 original research articles have been published on the topic (46, 47). A small study of 13 MS patients where half of them were treated with cyclosporine found an association where those treated had fewer cytotoxic CD8^+^ and more CD4^+^ T cells with less depression (48). Another small study examined MS patients with pre-existing depression versus those without depression. While they did not find difference in CD8^+^ T cell populations, the CD8 cells in depressed patients produced more TNF-α and interferon (IFN)-γ. This association held even when the authors controlled for disease modifying therapy and current functional status (49). This study as well another small study measured serum TNF-α and IFN-γ and found they were associated with chronic fatigue as well (50). Another small study of 47 MS patients found that IL-6 in CSF was independently associated with depression and fatigue. Furthermore, in a larger study with 249 healthy controls, 108 MS patients without depression, and 42 patients with MS and depression; serum IL-6 was highest in those with MS and depression. Interestingly, serum IL-6 is also a marker of depression in the general population, further supporting the idea that inflammation may be final common pathway to Brain FADE syndrome (51, 52). Finally, in small study of cognitive fog, there seemed to be an inverse relationship between T cell production of IFN-γ and fog; however, this study was not controlled for IFN-β therapy, the most common MS therapy, which is known to lower levels of IFN-γ-producing CD8+ T cells (49, 53). Despite the fact that every RCT in MS tests an immunomodulator only one of them used Brain FADE as an endpoint: Satralizumab (humanized antibody against IL-6) (54). The Satralizumab trial enrolled 83 patients with neuromyelitis optica, a type of MS. The authors found the Satralizumab arm had a reduced relapse rate, but no change in fatigue or depression. While a negative study for Brain FADE, at least this study provides definite causal evidence for the lack of involvement of IL-6 in fatigue and depression in this type of MS. More studies like the Satralizumab trial are required to determine causal roles for the many immunomodulatory drugs used to treat MS and Brain FADE. Taking a lesson from stroke, with utility weighted mRS, and the Patient Reported Outcome Measurement Information System (PROMIS) used to define postacute sequelae of SARS-CoV-2 infection (PASC); the patient’s input is critical to understand the relative importance of motor function versus Brain FADE.

## 4 Parkinson’s disease

Neurodegenerative diseases such as Parkinson’s disease have chronic inflammation that has been established in the glia (microglia, astrocytes, and neurons) of the brain in preclinical models, and serum inflammatory markers such as IL-2, IL-6, and TNF-α have been associated with depression and fatigue (55–58). Despite this association and the fact that over 50% of PD patients display some element of Brain FADE; no large scale, anti-inflammatory, RCTs have taken place in Parkinson’s disease to address this issue (59). 3 small trials have aimed to address inflammation through a variety of mechanisms. Azathioprine (AZA-PD) is phase II RCT that was begun in 2020 and aims to enroll 60 PD patients to assess gait and ataxia as primary outcomes along with inflammatory mediators as secondary outcomes, but not depression, fatigue, or fog (60). Similarly, a an RCT with the iron chelator, deferiprone, did not show any improvement in cognition or gait, but Brain FADE was not investigated (61). Finally, a general bcr-abl kinase inhibitor, nilotinib, was tried based on MPTP, pre-clinical models of PD that showed increased TREM-2 expression on microglia and decreased α-synuclein. TREM-2 is celebrated marker of microglial function that induces clustering of microglia around protein inclusions and is critical in the signal transduction required for the phagocytosis of these inclusions (62). The phase II study showed increased TREM-2 expression on CSF myeloid cells, increased serum dopamine, and decreased serum α-synuclein. While the study was preliminary, it remains to be seen if Brain FADE will be studied in future trials with the tyrosine kinase inhibitor (63).

## 5 Long COVID

When discussing Brain FADE syndrome, the infection of over half a billion people in the recent pandemic with SARS-CoV-2 (COVID-19) must not be overlooked (64). About 6% of patients with COVID-19 have symptoms that do not resolve for months or years, hence Long COVID (65). SARS-CoV-2 is a single-stranded positive-sense RNA virus with spike projections that emerge from the virions’ surface, a characteristic of the Coronaviridae family. Infection with coronavirus yields a significant inflammatory response stemming from both the innate TLR3 and cGAS-STING pathways, as well as the adaptive cytotoxic CD8^+^ T cells (66). Specific to SARS-CoV-2 are 4 critical structural elements of the virus: the spike, membrane, envelope, and nucleocapsid proteins. SARS-CoV-2 most likely infects patients through the cribriform plate and olfactory nerve, thus explaining the common symptoms of anosmia and ageusia. The olfactory nerve, or cranial nerve I, is the only cranial nerve that does not synapse before it enters the brain parenchyma. Given this method of infection, it is not surprising that SARS-CoV-2 would present with acute neurological symptoms like anosmia, headache, encephalitis, and ischemic stroke, or chronic ones such as impaired executive functions and fatigue (67).

Moreover, the fact that steroids proved to be such an effective treatment in COVID-19-induced acute respiratory distress syndrome (ARDS), provides a causal relationship between the inflammation and ARDS (68, 69). Long term outcomes after COVID infection, even those patients not ventilated, demonstrates a Brain FADE syndrome called postacute sequelae of SARS-CoV-2 infection (PASC), which affects 10%. A recent study of almost 14,000 patients that survived COVID, utilized the Patient Reported Outcome Measurement Information System (PROMIS) database to describe what has previously been called “long COVID.” Interestingly, patients defined PASC by 3 main criteria: fatigue, fog, and post-exertional malaise (4).

The main hypothesis put forth that might explain the persistent inflammation leading to Long COVID is persistence of the virus, found in the brain, gut, and lung parenchyma for up to 230 days after initial infection (70). Furthermore, the virus can persist in tissues despite negative whole blood and nasopharyngeal PCR tests. One team found increased SARS-CoV-2 proteins in extracellular vesicles derived from neurons and astrocytes circulating at higher levels in PASC patients than those that had completely recovered (71). These circulating proteins could have the ability to activate classic innate immune pathways in both macrophages and neutrophils to initiate anti-viral responses through TLR3 and the cGAS/STING pathway, both which heavily activate interferons and contribute to cytokine storm pathology (72). The persistent viral load and inflammation can be sensed by the vagus nerve, which has thousands of afferent projections from the organs in the body. This visceral inflammation is sensed by the vagus nerve and results in a sickness behavior like PASC due to glial activation and neuroinflammation (73–76). This was also demonstrated in a murine model of bacterial pneumonia where pulmonary inflammation resulted in the activation of nociceptive afferent neurons, a branch of the vagus nerve. Subsequently, vagal efferents to the lung suppressed neutrophil and T cell responses resulting in lethal pneumonia (77).

Besides the visceral sickness mediated by the vagus nerve that can lead to PASC or Brain FADE, a more direct pathophysiology exists based on spike protein binding affinity for amyloid, synuclein, and tau proteins. These protein inclusions in neurons and the cerebral interstitial fluid are thought to be involved in Alzheimer’s and Parkinson’s and Traumatic Brain Injury. Lending credence to this hypothesis, one group found that the incidence of new onset of Alzheimer’s diagnoses was significantly increased in older adults in the year following COVID infection Another autopsy found increased amyloid accumulation in the brains of patients with severe COVID illness younger than 60 years old (78, 79).

Further studies into these Brain FADE syndromes, and especially the brain-lung relationship via the vagus nerve are necessary in order to appropriately address and treat the chronic inflammatory component, possibly by vagotomy or vagus nerve stimulator.

## 6 Alzheimer’s disease

In Alzheimer’s disease (AD) the natural course of the disease leads to fog, but not necessarily fatigue and depression which can affect upwards of 40%. AD has been studied extensively with respect to pre-clinical models and clinical trials, but without significant emphasis on depression and fatigue as an endpoint. Attempts at treating cyclo-oxygenase induced inflammation in the aged population failed to reduce depression (80, 81). Alzheimer studies are unique in that a multitude of anti-inflammatory agents are being tested, ranging from herbal remedies like resveratrol to bromodomain epigenetic proteins (BET) to Apoϵ mimetics (82–84). Of these studies, only the MARBLE study, using an Apo mimetic, is specifically studying depression and fatigue in perioperative cognitive dysfunction. So even in Alzheimer’s disease where depression can be seen in 90% of the population and fatigue and sleep disorders in 70%, these are not primary outcomes that are studied as a result of chronic inflammation (85).

## 7 Traumatic brain injury

Finally, traumatic brain injury (TBI), is perhaps the only disease, where Brain FADE syndrome is synonymous with the post-traumatic stress disorder (PTSD) moniker, apart from the etiologies that lead to these syndromes. While PTSD follows TBI, the mechanism by which one causes the other is not defined; as opposed to Brain FADE syndrome, where the mechanism is hypothesized to be chronic inflammation, regardless of the initial insult. In civilian populations, the frequency of PTSD is 18.6% after 12 months. In military populations, the frequency is reported to be upwards of 48.2%; however, this depends on the war as well as the variety of prior descriptions of this syndrome including, shell shock, disordered action of the heart, effort syndrome, effects of Agent Orange, and Gulf War syndrome to name a few (86, 87). From the TRACK-TBI study of civilians, frequencies of PTSD and MDD were relatively common, with 6-month rates ranging from 9% to 19% (88). Moreover, this study defined the population at greatest risk for PTSD and MDD after mild TBI: less education, being black, self-reported psychiatric history, and injury resulting from assault or other violence. These critical epidemiological studies are exactly what is needed in every neurological disease to carefully define risk factors for Brain FADE syndrome so that the chronic inflammation component can be studied in these at-risk populations.

Similar to previous neurological diseases, very few RCTs have been conducted to address Brain FADE syndrome or PTSD in TBI patients. Unlike other neurological disease, chronic inflammation is recognized as a critical factor in PTSD, as a tertiary phase of injury (89). The MRC Crash trial TBI patients were treated with high dose steroids for 48 hours and showed no improvement in outcomes (90). Consistent with the concept of Brain FADE and tertiary injury after TBI, low dose steroids have shown to improve PTSD (91, 92). Progesterone and erythropoietin both failed to improve outcomes in TBI, even though both showed reproducible, marked effects in rodent models of TBI (93, 94). Mifepristone, on the other hand, was specifically targeted against PTSD in war fighters, but also failed to show any efficacy (95). While all these therapies circuitously address Brain FADE syndrome or PTSD in TBI, a small, open label trial using etanercept, a soluble antibody to TNF-α, showed significant improvement across all domains of Brain FADE syndrome (96). Further RCTs that specifically target chronic inflammation are necessary. By and large the translational validity of treatment in TBI from rodent to human has been dismal. This may be due to several factors, among the most important could be that quadrupeds have a thicker cortex that is in line with their brainstem, whereas humans have a thinner cortex that is perpendicular to their brainstem with much more with matter, making diffuse axonal injury frequent. Furthermore, any TBI model requires sedation before the impact, the most common being ketamine or isoflurane, both of which have neuroprotective effects (89). The variety of traumatic brain injury mechanisms in humans are also difficult to replicate from improvised explosive devices, to boxing, to high-speed motor vehicle accidents, to violent attacks from our own species. Because so much of TBI is not only the mechanism of injury, but the circumstances surrounding the trauma; it is not surprising that researchers cannot capture this with a simple controlled cortical impact model. Even if the “meta-data” surrounding the trauma, and the mechanism of injury could be accurately replicated, the measurement of fatigue, fog, and depression would prove difficult in a rodent. Future models of traumatic brain injury need to be applied to one mechanism of injury that most closely resembles a CCI, such as blunt force trauma to the head, and only large mammals capable of easily displaying depression or fatigue should be used as a first step toward a “win” in TBI therapeutics.

## 8 Conclusion

The role of inflammation as well as targeting inflammation alone may be a critical factor for the treatment of virtually all neurological disease, but these classical “psychiatric endpoints” need to be embraced by neurologists and neuroscientists, both in the lab and the clinic. A focus on the final common pathway will likely involve microglia, Toll like receptors, T lymphocytes, and IL-6. The approach to Brain FADE syndrome needs to be similar to that seen in oncology, where multiple targets are chosen along a common pathway, such as the treatment of HER2-positive breast cancer. Here, combination therapies attack the cancer along multiple pathways to enhance efficacy, manage resistance, and minimize side effects. Monoclonal antibodies against the HER2 protein inhibit growth of the tumor through this pathway; trastuzumab, targets one part of the HER2 protein and pertuzumab another. With growth inhibited, “-cidal” agents that kill cancer cells are then used, like paclitaxel. Finally, if the cancer is estrogen receptor positive, an aromatase inhibitor like tamoxifen can be used to maximize inhibition of all trophic pathways for the cancer (97).

As the tissue resident macrophage of the brain, microglia and its response to PAMPs and DAMPs will likely be critical in phase I of a pharmaceutical attack, such as a TLR4 aptamer. However, there are pathways that will allow for autonomous activation of lymphocytes a sustained immune response, independent of antigen presenting cell activation. Targeting T cells (Teriflunomide) and IL-6 (Satralizumab) would allow for a phase II disruption, hopefully impeding the recruitment of adaptive immunity to the propagation of chronic inflammation. Drug combination regimens are critical to the treatment of virtually every cancer; the application of a multi-targeted, phased approach could yield amazing results against Brain FADE syndrome and beyond.

## Author contributions

KH: Writing – original draft, Writing – review & editing. TJ: Conceptualization, Supervision, Writing – review & editing.

## References

[1] Parikh NS Merkler AE Iadecola C. Inflammation, autoimmunity, infection, and stroke: epidemiology and lessons from therapeutic intervention. Stroke (2020) 51:711–8. doi: 10.1161/STROKEAHA.119.024157 PMC7041866 32078460

[2] Annoni J-M Staub F Bogousslavsky J Brioschi A. Frequency, characterisation and therapies of fatigue after stroke. Neurol Sci Off J Ital Neurol Soc Ital Soc Clin Neurophysiol (2008) 29 Suppl 2:S244–246. doi: 10.1007/s10072-008-0951-0 18690506

[3] Beckerman H Eijssen IC van Meeteren J Verhulsdonck MC de Groot V. Fatigue profiles in patients with multiple sclerosis are based on severity of fatigue and not on dimensions of fatigue. Sci Rep (2020) 10:4167. doi: 10.1038/s41598-020-61076-1 32139797 PMC7058058

[4] Thaweethai T Jolley SE Karlson EW Levitan EB Levy B McComsey GA. Development of a definition of postacute sequelae of SARS-CoV-2 infection. JAMA (2023). doi: 10.1001/jama.2023.8823 PMC10214179 37278994

[5] Chollet F Tardy J Albucher J-F Thalamas C Berard E Lamy C. Fluoxetine for motor recovery after acute ischaemic stroke (FLAME): a randomised placebo-controlled trial. Lancet Neurol (2011) 10:123–30. doi: 10.1016/S1474-4422(10)70314-8 21216670

[6] Yi ZM Liu F Zhai SD. Fluoxetine for the prophylaxis of poststroke depression in patients with stroke: a meta-analysis. Int J Clin Pract (2010) 64:1310–7. doi: 10.1111/j.1742-1241.2010.02437.x 20653802

[7] Effects of fluoxetine on functional outcomes after acute stroke (FOCUS): a pragmatic, double-blind, randomised, controlled trial. Lancet Lond Engl (2019) 393:265–74. doi: 10.1016/S0140-6736(18)32823-X PMC6336936 30528472

[8] EFFECTS Trial Collaboration. Safety and efficacy of fluoxetine on functional recovery after acute stroke (EFFECTS): a randomised, double-blind, placebo-controlled trial. Lancet Neurol (2020) 19:661–9. doi: 10.1016/S1474-4422(20)30219-2 32702335

[9] AFFINITY Trial Collaboration. Safety and efficacy of fluoxetine on functional outcome after acute stroke (AFFINITY): a randomised, double-blind, placebo-controlled trial. Lancet Neurol (2020) 19:651–60. doi: 10.1016/S1474-4422(20)30207-6 32702334

[10] Goldstein LB Amarenco P Zivin J Messig M Altafullah I Callahan A. Statin treatment and stroke outcome in the stroke prevention by aggressive reduction in cholesterol levels (SPARCL) trial. Stroke (2009) 40:3526–31. doi: 10.1161/STROKEAHA.109.557330 19745172

[11] Wium-Andersen IK Wium-Andersen MK Jørgensen MB Osler M. Anti-inflammatory treatment and risk for depression after first-time stroke in a cohort of 147 487 Danish patients. J Psychiatry Neurosci (2017) 42:320–30. doi: 10.1503/jpn160244 PMC5573574 28632121

[12] Kang J-H Kao L-T Lin H-C Tsai M-C Chung S-D. Statin use increases the risk of depressive disorder in stroke patients: A population-based study. J Neurol Sci (2015) 348:89–93. doi: 10.1016/j.jns.2014.11.013 25483831

[13] Kim J-M. A prospective study of statin use and poststroke depression. J Clin Psychopharmacol (2014) 34:72. doi: 10.1097/JCP.0000000000000051 24304857

[14] Spalletta G Cravello L Imperiale F Salani F Bossù P Picchetto L. Neuropsychiatric symptoms and interleukin-6 serum levels in acute stroke. J Neuropsychiatry Clin Neurosci (2013) 25:255–63. doi: 10.1176/appi.neuropsych.12120399 24247852

[15] Yang L Zhang Z Sun D Xu Z Zhang X Li L. The serum interleukin-18 is a potential marker for development of post-stroke depression. Neurol Res (2010) 32:340–6. doi: 10.1179/016164110X12656393665080 20482998

[16] Lees KR Zivin JA Ashwood T Davalos A Davis SM Diener H-C. NXY-059 for acute ischemic stroke. N Engl J Med (2006) 354:588–600. doi: 10.1056/NEJMoa052980 16467546

[17] Shuaib A Lees KR Lyden P Grotta J Davalos A Davis SM. NXY-059 for the treatment of acute ischemic stroke. N Engl J Med (2007) 357:562–71. doi: 10.1056/NEJMoa070240 17687131

[18] Stroke Therapy Academic Industry Roundtable (STAIR). Recommendations for standards regarding preclinical neuroprotective and restorative drug development. Stroke (1999) 30:2752–8. doi: 10.1161/01.STR.30.12.2752 10583007

[19] Fisher M Feuerstein G Howells DW Hurn PD Kent TA Savitz SI. Update of the stroke therapy academic industry roundtable preclinical recommendations. Stroke J Cereb Circ (2009) 40:2244–50. doi: 10.1161/STROKEAHA.108.541128 PMC2888275 19246690

[20] Hill MD Goyal M Menon BK Nogueira RG McTaggart RA Demchuk AM. Efficacy and safety of nerinetide for the treatment of acute ischaemic stroke (ESCAPE-NA1): a multicentre, double-blind, randomised controlled trial. Lancet Lond Engl (2020) 395:878–87. doi: 10.1016/S0140-6736(20)30258-0 32087818

[21] Sattler R Xiong Z Lu WY Hafner M MacDonald JF Tymianski M. Specific coupling of NMDA receptor activation to nitric oxide neurotoxicity by PSD-95 protein. Science (1999) 284:1845–8. doi: 10.1126/science.284.5421.1845 10364559

[22] Aarts M Liu Y Liu L Besshoh S Arundine M Gurd JW. Treatment of ischemic brain damage by perturbing NMDA receptor- PSD-95 protein interactions. Science (2002) 298:846–50. doi: 10.1126/science.1072873 12399596

[23] Elkins J Veltkamp R Montaner J Johnston SC Singhal AB Becker K. Safety and efficacy of natalizumab in patients with acute ischaemic stroke (ACTION): a randomised, placebo-controlled, double-blind phase 2 trial. Lancet Neurol (2017) 16:217–26. doi: 10.1016/S1474-4422(16)30357-X 28229893

[24] Polman CH O’Connor PW Havrdova E Hutchinson M Kappos L Miller DH. A randomized, placebo-controlled trial of natalizumab for relapsing multiple sclerosis. N Engl J Med (2006) 354:899–910. doi: 10.1056/NEJMoa044397 16510744

[25] Rudick RA Stuart WH Calabresi PA Confavreux C Galetta SL Radue E-W. Natalizumab plus interferon beta-1a for relapsing multiple sclerosis. N Engl J Med (2006) 354:911–23. doi: 10.1056/NEJMoa044396 16510745

[26] Penner I-K Sivertsdotter EC Celius EG Fuchs S Schreiber K Berkö S. Improvement in fatigue during natalizumab treatment is linked to improvement in depression and day-time sleepiness. Front Neurol (2015) 6:18. doi: 10.3389/fneur.2015.00018 25755648 PMC4337367

[27] Planche V Moisset X Morello R Dumont E Gibelin M Charré-Morin J. Improvement of quality of life and its relationship with neuropsychiatric outcomes in patients with multiple sclerosis starting treatment with natalizumab: A 3-year follow-up multicentric study. J Neurol Sci (2017) 382:148–54. doi: 10.1016/j.jns.2017.10.008 29111011

[28] Rorsman I Petersen C Nilsson PC. Cognitive functioning following one-year natalizumab treatment: A non-randomized clinical trial. Acta Neurol Scand (2018) 137:117–24. doi: 10.1111/ane.12833 28901547

[29] Svenningsson A Falk E Celius EG Fuchs S Schreiber K Berkö S. Natalizumab treatment reduces fatigue in multiple sclerosis. Results from the TYNERGY trial; a study in the real life setting. PloS One (2013) 8:e58643. doi: 10.1371/journal.pone.0058643 23555589 PMC3605436

[30] O’Neill LAJ Bowie AG. The family of five: TIR-domain-containing adaptors in Toll-like receptor signalling. Nat Rev Immunol (2007) 7:353–64. doi: 10.1038/nri2079 17457343

[31] Akamatsu Y Pagan VA Hanafy KA. The role of TLR4 and HO-1 in neuroinflammation after subarachnoid hemorrhage. J Neurosci Res (2020) 98:549–56. doi: 10.1002/jnr.24515 PMC6980436 31468571

[32] Hanafy KA. The role of microglia and the TLR4 pathway in neuronal apoptosis and vasospasm after subarachnoid hemorrhage. J Neuroinflamm (2013) 10:83. doi: 10.1186/1742-2094-10-83 PMC3750560 23849248

[33] Islam R Vrionis F Hanafy KA. Microglial TLR4 is critical for neuronal injury and cognitive dysfunction in subarachnoid hemorrhage. Neurocrit Care (2022) 37:761–9. doi: 10.1007/s12028-022-01552-w PMC9672010 35778649

[34] Sansing LH Harris TH Welsh FA Kasner SE Hunter CA Kariko K. Toll-like receptor 4 contributes to poor outcome after intracerebral hemorrhage. Ann Neurol (2011) 70:646–56. doi: 10.1002/ana.22528 PMC3671585 22028224

[35] Caso JR Pradillo JM Hurtado O Lorenzo P Moro MA Lizasoain I. Toll-like receptor 4 is involved in brain damage and inflammation after experimental stroke. Circulation (2007) 115:1599–608. doi: 10.1161/CIRCULATIONAHA.106.603431 17372179

[36] Fernández G Moraga A Cuartero MI García-Culebras A Peña-Martínez C Pradillo JM. TLR4-binding DNA aptamers show a protective effect against acute stroke in animal models. Mol Ther J Am Soc Gene Ther (2018) 26:2047–59. doi: 10.1016/j.ymthe.2018.05.019 PMC6094477 29910175

[37] Hernández-Jiménez M Martín-Vílchez S Ochoa D Mejía-Abril G Román M Camargo-Mamani P. First-in-human phase I clinical trial of a TLR4-binding DNA aptamer, ApTOLL: Safety and pharmacokinetics in healthy volunteers. Mol Ther Nucleic Acids (2022) 28:124–35. doi: 10.1016/j.omtn.2022.03.005 PMC8938885 35402075

[38] Hernández-Jiménez M Abad-Santos F Cotgreave I Gallego J Jilma B Flores A. APRIL: A double-blind, placebo-controlled, randomized, Phase Ib/IIa clinical study of ApTOLL for the treatment of acute ischemic stroke. Front Neurol (2023) 14:1127585. doi: 10.3389/fneur.2023.1127585 36908619 PMC9999729

[39] Grotta JC Yamal J-M Parker SA Rajan SS Gonzales NR Jones WJ. Prospective, multicenter, controlled trial of mobile stroke units. N Engl J Med (2021) 385:971–81. doi: 10.1056/NEJMoa2103879 34496173

[40] Krupp LB Serafin DJ Christodoulou C. Multiple sclerosis-associated fatigue. Expert Rev Neurother (2010) 10:1437–47. doi: 10.1586/ern.10.99 20819014

[41] Chiaravalloti ND DeLuca J. Cognitive impairment in multiple sclerosis. Lancet Neurol (2008) 7:1139–51. doi: 10.1016/S1474-4422(08)70259-X 19007738

[42] Feinstein A Magalhaes S Richard J-F Audet B Moore C. The link between multiple sclerosis and depression. Nat Rev Neurol (2014) 10:507–17. doi: 10.1038/nrneurol.2014.139 25112509

[43] Oliva Ramirez A Keenan A Kalau O Worthington E Cohen L Singh S. Prevalence and burden of multiple sclerosis-related fatigue: a systematic literature review. BMC Neurol (2021) 21:468. doi: 10.1186/s12883-021-02396-1 34856949 PMC8638268

[44] Cortese R Carotenuto A Di Filippo M Lanzillo R. Editorial: cognition in multiple sclerosis. Front Neurol (2021) 12:751687. doi: 10.3389/fneur.2021.751687 34526964 PMC8435676

[45] Marrie RA Reingold S Cohen J Stuve O Trojano M Sorensen PS. The incidence and prevalence of psychiatric disorders in multiple sclerosis: a systematic review. Mult. Scler. Houndmills Basingstoke Engl (2015) 21:305–17. doi: 10.1177/1352458514564487 PMC4429164 25583845

[46] Chitnis T Vandercappellen J King M Brichetto G. Symptom interconnectivity in multiple sclerosis: A narrative review of potential underlying biological disease processes. Neurol Ther (2022) 11:1043–70. doi: 10.1007/s40120-022-00368-2 PMC9338216 35680693

[47] Dantzer R O’Connor JC Freund GG Johnson RW Kelley KW. From inflammation to sickness and depression: when the immune system subjugates the brain. Nat Rev Neurosci (2008) 9:46–56. doi: 10.1038/nrn2297 18073775 PMC2919277

[48] Foley FW Traugott U LaRocca NG Smith CR Perlman KR Caruso LS. A prospective study of depression and immune dysregulation in multiple sclerosis. Arch Neurol (1992) 49:238–44. doi: 10.1001/archneur.1992.00530270052018 1536625

[49] Gold SM Krüger S Ziegler KJ Krieger T Schulz K-H Otte C. Endocrine and immune substrates of depressive symptoms and fatigue in multiple sclerosis patients with comorbid major depression. J Neurol Neurosurg Psychiatry (2011) 82:814–8. doi: 10.1136/jnnp.2010.230029 21296901

[50] Heesen C Nawrath L Reich C Bauer N Schulz K-H Gold SM. Fatigue in multiple sclerosis: an example of cytokine mediated sickness behaviour? J Neurol Neurosurg Psychiatry (2006) 77:34–9. doi: 10.1136/jnnp.2005.065805 PMC2117393 16361589

[51] Liu Y Ho RC-M Mak A. Interleukin (IL)-6, tumour necrosis factor alpha (TNF-α) and soluble interleukin-2 receptors (sIL-2R) are elevated in patients with major depressive disorder: a meta-analysis and meta-regression. J Affect. Disord (2012) 139:230–9. doi: 10.1016/j.jad.2011.08.003 21872339

[52] Valkanova V Ebmeier KP Allan CL. CRP, IL-6 and depression: a systematic review and meta-analysis of longitudinal studies. J Affect Disord (2013) 150:736–44. doi: 10.1016/j.jad.2013.06.004 23870425

[53] Heesen C Schulz KH Fiehler J Von der Mark U Otte C Jung R. Correlates of cognitive dysfunction in multiple sclerosis. Brain Behav Immun (2010) 24:1148–55. doi: 10.1016/j.bbi.2010.05.006 20621641

[54] Yamamura T Kleiter I Fujihara K Palace J Greenberg B Zakrzewska-Pniewska B. Trial of satralizumab in neuromyelitis optica spectrum disorder. N Engl J Med (2019) 381:2114–24. doi: 10.1056/NEJMoa1901747 31774956

[55] Bu X-L Wang X Xiang Y Shen L-L Wang Q-H Liu Y-H. The association between infectious burden and Parkinson’s disease: A case-control study. Parkinsonism Relat Disord (2015) 21:877–81. doi: 10.1016/j.parkreldis.2015.05.015 26037459

[56] Chen K Wang H Ilyas I Mahmood A Hou L. Microglia and astrocytes dysfunction and key neuroinflammation-based biomarkers in Parkinson’s disease. Brain Sci (2023) 13:634. doi: 10.3390/brainsci13040634 37190599 PMC10136556

[57] Mogi M Harada M Kondo T Riederer P Nagatsu T. Interleukin-2 but not basic fibroblast growth factor is elevated in parkinsonian brain. Short communication. J Neural Transm Vienna Austria 1996 (1996) 103:1077–81. doi: 10.1007/BF01291792 9013395

[58] Mogi M Harada M Riederer P Narabayashi H Fujita K Nagatsu T. Tumor necrosis factor-alpha (TNF-alpha) increases both in the brain and in the cerebrospinal fluid from parkinsonian patients. Neurosci Lett (1994) 165:208–10. doi: 10.1016/0304-3940(94)90746-3 8015728

[59] Allain H Schuck S Mauduit N. Depression in Parkinson’s disease. BMJ (2000) 320:1287–8. doi: 10.1136/bmj.320.7245.1287 PMC1127292 10807601

[60] Greenland JC Cutting E Kadyan S Bond S Chhabra A Williams-Gray CH. Azathioprine immunosuppression and disease modification in Parkinson’s disease (AZA-PD): a randomised double-blind placebo-controlled phase II trial protocol. BMJ Open (2020) 10:e040527. doi: 10.1136/bmjopen-2020-040527 PMC7684836 33234645

[61] Martin-Bastida A Ward RJ Newbould R Piccini P Sharp D Kabba C. Brain iron chelation by deferiprone in a phase 2 randomised double-blinded placebo controlled clinical trial in Parkinson’s disease. Sci Rep (2017) 7:1398. doi: 10.1038/s41598-017-01402-2 28469157 PMC5431100

[62] Gratuze M Leyns CEG Holtzman DM. New insights into the role of TREM2 in Alzheimer’s disease. Mol Neurodegener (2018) 13:66. doi: 10.1186/s13024-018-0298-9 30572908 PMC6302500

[63] Pagan FL Hebron ML Wilmarth B Torres-Yaghi Y Lawler A Mundel EE. Pharmacokinetics and pharmacodynamics of a single dose Nilotinib in individuals with Parkinson’s disease. Pharmacol Res Perspect (2019) 7:e00470. doi: 10.1002/prp2.470 30906562 PMC6412143

[64] WHO Coronavirus (COVID-19) dashboard. Available at: https://covid19.who.int.

[65] Proal AD VanElzakker MB. Long COVID or post-acute sequelae of COVID-19 (PASC): an overview of biological factors that may contribute to persistent symptoms. Front Microbiol (2021) 12. doi: 10.3389/fmicb.2021.698169 PMC8260991 34248921

[66] Khanmohammadi S Rezaei N. Role of Toll-like receptors in the pathogenesis of COVID-19. J Med Virol (2021) 93:2735–9. doi: 10.1002/jmv.26826 PMC8014260 33506952

[67] Zaa CA Espitia C Reyes-Barrera KL An Z Velasco-Velázquez MA. Neuroprotective agents with therapeutic potential for COVID-19. Biomolecules (2023) 13:1585. doi: 10.3390/biom13111585 38002267 PMC10669388

[68] RECOVERY Collaborative Group Horby P Lim WS Emberson JR Mafham M Bell JL. Dexamethasone in hospitalized patients with covid-19. N Engl J Med (2021) 384:693–704. doi: 10.1056/NEJMoa2021436 32678530 PMC7383595

[69] Tomazini BM Maia IS Cavalcanti AB Berwanger O Rosa RG Veiga VC. Effect of dexamethasone on days alive and ventilator-free in patients with moderate or severe acute respiratory distress syndrome and COVID-19: the CoDEX randomized clinical trial. JAMA (2020) 324:1307–16. doi: 10.1001/jama.2020.17021 PMC7489411 32876695

[70] Stein SR Ramelli SC Grazioli A Chung J-Y Singh M Yinda CK. SARS-CoV-2 infection and persistence in the human body and brain at autopsy. Nature (2022) 612:758–63. doi: 10.1038/s41586-022-05542-y PMC9749650 36517603

[71] Peluso MJ Deeks SG Mustapic M Kapogiannis D Henrich TJ Lu S. SARS-CoV-2 and mitochondrial proteins in neural-derived exosomes of COVID-19. Ann Neurol (2022) 91:772–81. doi: 10.1002/ana.26350 PMC9082480 35285072

[72] Chilosi M Doglioni C Ravaglia C Piciucchi S Dubini A Stefanizzi L. COVID-19. Biology, pathophysiology, and immunology: a pathologist view. Pathologica (2023) 115:248–56. doi: 10.32074/1591-951X-954 PMC10699332 38054899

[73] McCusker RH Kelley KW. Immune-neural connections: how the immune system’s response to infectious agents influences behavior. J Exp Biol (2013) 216:84–98. doi: 10.1242/jeb.073411 23225871 PMC3515033

[74] Goehler LE Gaykema RPA Opitz N Reddaway R Badr N Lyte M. Activation in vagal afferents and central autonomic pathways: early responses to intestinal infection with Campylobacter jejuni. Brain. Behav Immun (2005) 19:334–44. doi: 10.1016/j.bbi.2004.09.002 15944073

[75] Woo MS Shafiq M Fitzek A Dottermusch M Altmeppen H Mohammadi B. Vagus nerve inflammation contributes to dysautonomia in COVID-19. Acta Neuropathol (Berl) (2023) 146:387–94. doi: 10.1007/s00401-023-02612-x PMC10412500 37452829

[76] Matschke J Lütgehetmann M Hagel C Sperhake JP Schröder AS Edler C. Neuropathology of patients with COVID-19 in Germany: a post-mortem case series. Lancet Neurol (2020) 19:919–29. doi: 10.1016/S1474-4422(20)30308-2 PMC7535629 33031735

[77] Baral P Umans BD Li L Wallrapp A Bist M Kirschbaum T. Nociceptor sensory neurons suppress neutrophil and γδ T cell responses in bacterial lung infections and lethal pneumonia. Nat Med (2018) 24:417–26. doi: 10.1038/nm.4501 PMC6263165 29505031

[78] Proal AD VanElzakker MB Aleman S Bach K Boribong BP Buggert M. SARS-CoV-2 reservoir in post-acute sequelae of COVID-19 (PASC). Nat Immunol (2023) 24:1616–27. doi: 10.1038/s41590-023-01601-2 37667052

[79] Idrees D Kumar V. SARS-CoV-2 spike protein interactions with amyloidogenic proteins: Potential clues to neurodegeneration. Biochem Biophys Res Commun (2021) 554:94–8. doi: 10.1016/j.bbrc.2021.03.100 PMC7988450 33789211

[80] Berk M Agustini B Woods RL Nelson MR Shah RC Reid CM. Effects of aspirin on the long-term management of depression in older people: a double-blind randomised placebo-controlled trial. Mol Psychiatry (2021) 26:5161–70. doi: 10.1038/s41380-021-01020-5 PMC8313623 33504953

[81] Fields C Drye L Vaidya V Lyketsos C ADAPT Research Group. Celecoxib or naproxen treatment does not benefit depressive symptoms in persons age 70 and older: findings from a randomized controlled trial. Am J Geriatr Psychiatry Off J Am Assoc Geriatr Psychiatry (2012) 20:505–13. doi: 10.1097/JGP.0b013e318227f4da PMC3209494 21775876

[82] Moussa C Hebron M Huang X Ahn J Rissman RA Aisen PS. Resveratrol regulates neuro-inflammation and induces adaptive immunity in Alzheimer’s disease. J Neuroinflamm (2017) 14:1. doi: 10.1186/s12974-016-0779-0 PMC5234138 28086917

[83] Cummings J Schwartz GG Nicholls SJ Khan A Halliday C Toth PP. Cognitive effects of the BET protein inhibitor apabetalone: A prespecified montreal cognitive assessment analysis nested in the BETonMACE randomized controlled trial. J Alzheimers Dis JAD (2021) 83:1703–15. doi: 10.3233/JAD-210570 PMC8609701 34459400

[84] VanDusen KW Eleswarpu S Moretti EW Devinney MJ Crabtree DM Laskowitz DT. The MARBLE study protocol: modulating apoE signaling to reduce brain inflammation, deLirium, and postopErative cognitive dysfunction. J Alzheimers Dis JAD (2020) 75:1319–28. doi: 10.3233/JAD-191185 PMC7923142 32417770

[85] Fong KN Ge X Ting KH Wei M Cheung H. The effects of light therapy on sleep, agitation and depression in people with dementia: A systematic review and meta-analysis of randomized controlled trials. Am J Alzheimers Dis Other Demen (2023) 38:15333175231160682. doi: 10.1177/15333175231160682 36924042 PMC10578524

[86] Iljazi A Ashina H Al-Khazali HM Lipton RB Ashina M Schytz HW. Post-traumatic stress disorder after traumatic brain injury-A systematic review and meta-analysis. Neurol Sci Off J Ital Neurol Soc Ital Soc Clin Neurophysiol (2020) 41:2737–46. doi: 10.1007/s10072-020-04458-7 32415640

[87] Jones E Wessely S. War syndromes: the impact of culture on medically unexplained symptoms. Med Hist (2005) 49:55–78. doi: 10.1017/S0025727300008280 15730130 PMC1088250

[88] Stein MB Jain S Giacino JT Levin H Dikmen S Nelson LD. Risk of posttraumatic stress disorder and major depression in civilian patients after mild traumatic brain injury: A TRACK-TBI study. JAMA Psychiatry (2019) 76:249–58. doi: 10.1001/jamapsychiatry.2018.4288 PMC6439818 30698636

[89] Jacquens A Needham EJ Zanier ER Degos V Gressens P Menon D. Neuro-inflammation modulation and post-traumatic brain injury lesions: from bench to bed-side. Int J Mol Sci (2022) 23:11193. doi: 10.3390/ijms231911193 36232495 PMC9570205

[90] Roberts I Yates D Sandercock P Farrell B Wasserberg J Lomas G. Effect of intravenous corticosteroids on death within 14 days in 10008 adults with clinically significant head injury (MRC CRASH trial): randomised placebo-controlled trial. Lancet Lond Engl (2004) 364:1321–8. doi: 10.1016/S0140-6736(04)17188-2 15474134

[91] Yehuda R Bierer LM Pratchett LC Lehrner A Koch EC Van Manen JA. Cortisol augmentation of a psychological treatment for warfighters with posttraumatic stress disorder: Randomized trial showing improved treatment retention and outcome. Psychoneuroendocrinology (2015) 51:589–97. doi: 10.1016/j.psyneuen.2014.08.004 25212409

[92] Lehrner A Hildebrandt T Bierer LM Flory JD Bader HN Makotkine I. A randomized, double-blind, placebo-controlled trial of hydrocortisone augmentation of Prolonged Exposure for PTSD in U.S. combat veterans. Behav Res Ther (2021) 144:103924. doi: 10.1016/j.brat.2021.103924 34298438

[93] Wright DW Yeatts SD Silbergleit R Palesch YY Hertzberg VS Frankel M. Very early administration of progesterone for acute traumatic brain injury. N Engl J Med (2014) 371:2457–66. doi: 10.1056/NEJMoa1404304 PMC4303469 25493974

[94] Nichol A French C Little L Haddad S Presneill J Arabi Y. Erythropoietin in traumatic brain injury (EPO-TBI): a double-blind randomised controlled trial. Lancet Lond Engl (2015) 386:2499–506. doi: 10.1016/S0140-6736(15)00386-4 26452709

[95] Golier JA Li X Bizien M Hurley RA Bechard BW Kimbrell T. Efficacy and safety of mifepristone in the treatment of male US veterans with posttraumatic stress disorder: A phase 2a randomized clinical trial. JAMA Netw Open (2023) 6:e2310223. doi: 10.1001/jamanetworkopen.2023.10223 37159200 PMC10170341

[96] Tobinick E Kim NM Reyzin G Rodriguez-Romanacce H DePuy V. Selective TNF inhibition for chronic stroke and traumatic brain injury: an observational study involving 629 consecutive patients treated with perispinal etanercept. CNS Drugs (2012) 26:1051–70. doi: 10.1007/s40263-012-0013-2 23100196

[97] Jerzak KJ Bouganim N Brezden-Masley C Edwards S Gelmon K Henning J-W. HR+/HER2– advanced breast cancer treatment in the first-line setting: expert review. Curr Oncol (2023) 30:5425–47. doi: 10.3390/curroncol30060411 PMC10297170 37366894

